# Evidence of Drug–Nutrient Interactions with Chronic Use of Commonly Prescribed Medications: An Update

**DOI:** 10.3390/pharmaceutics10010036

**Published:** 2018-03-20

**Authors:** Emily S. Mohn, Hua J. Kern, Edward Saltzman, Susan H. Mitmesser, Diane L. McKay

**Affiliations:** 1 Jean Mayer USDA Human Nutrition Research Center on Aging, and Friedman School of Nutrition Science and Policy, Tufts University, Boston, MA 02111, USA; emily.mohn@tufts.edu (E.S.M.); edward.saltzman@tufts.edu (E.S.); 2 Nutrition & Scientific Affairs, Nature’s Bounty Co., Ronkonkoma, NY 11779, USA; carinakern@nbty.com (H.J.K.); susanmitmesser@nbty.com (S.H.M.)

**Keywords:** drug–nutrient interaction, micronutrient deficiencies, nutrient inadequacies, multivitamin, dietary supplement

## Abstract

The long-term use of prescription and over-the-counter drugs can induce subclinical and clinically relevant micronutrient deficiencies, which may develop gradually over months or even years. Given the large number of medications currently available, the number of research studies examining potential drug–nutrient interactions is quite limited. A comprehensive, updated review of the potential drug–nutrient interactions with chronic use of the most often prescribed medications for commonly diagnosed conditions among the general U.S. adult population is presented. For the majority of the interactions described in this paper, more high-quality intervention trials are needed to better understand their clinical importance and potential consequences. A number of these studies have identified potential risk factors that may make certain populations more susceptible, but guidelines on how to best manage and/or prevent drug-induced nutrient inadequacies are lacking. Although widespread supplementation is not currently recommended, it is important to ensure at-risk patients reach their recommended intakes for vitamins and minerals. In conjunction with an overall healthy diet, appropriate dietary supplementation may be a practical and efficacious way to maintain or improve micronutrient status in patients at risk of deficiencies, such as those taking medications known to compromise nutritional status. The summary evidence presented in this review will help inform future research efforts and, ultimately, guide recommendations for patient care.

## 1. Introduction

The long-term use of prescription and over the counter (OTC) drugs can induce subclinical and clinically relevant micronutrient deficiencies which may develop gradually over months or even years. Unfortunately, nutrient deficiencies seldom present as classically described and, with the exception of the most common micronutrient issues, many health care providers are not knowledgeable about micronutrient deficiency or excess. This may lead to erroneous attribution of deficiency states to a disease state or the aging process itself [1] and may delay diagnosis. Drug-induced micronutrient depletion may be the origin of otherwise unexplained symptoms, some of which might influence medication compliance [2].

Drug–nutrient interactions are defined as physical, chemical, physiologic, or pathophysiologic relationships between a drug and a nutrient, and typically involve multiple factors [3,4]. Drugs can influence food intake, nutrient digestion, absorption, distribution, metabolism to active forms, function, catabolism and excretion [5,6]. Additionally, the presence of compound-specific transport proteins, receptors, and enzymes in different tissues alters the pattern and location of where drugs and nutrients interact, creating scores of possible tissue-specific interactions [4,5], and makes prediction of clinical effects difficult. Ethanol and tobacco also influence micronutrients in ways similar to drugs, but discussion of this is beyond the scope of this review.

According to the National Ambulatory Care Service Survey released by the National Center for Health Statistics, the chronic conditions most often present in patients >45 years that require chronic drug use include hypertension, hyperlipidemia, arthritis, diabetes, depression, asthma, ischemic heart disease, and chronic obstructive pulmonary disease (COPD) [7]. Data from the National Health Interview Survey showed the prevalence of hypertension among adults was 29.0% in 2011–2014 and increased with age [8]. Furthermore 12% of individuals aged 45–64 years, and 29.4% aged ≥65 years were diagnosed with heart disease (all types), 27.4% of all adults aged ≥20 years had hypercholesteremia, and 11.9% had diabetes. Although only 3.4% of adults were reported to have serious psychological distress, including depression, these adults were more likely to have COPD, heart disease, and diabetes [9]. This suggests there is a higher prevalence of depression in individuals with chronic disease. A substantial number of adults reported having conditions that may warrant the use of nonsteroidal anti-inflammatory drugs (NSAIDS) or corticosteroids, including osteoarthritis, which is the most common cause of disability in older adults, as well as low back pain (28.1% of adults), severe headache or migraine (15.3%), and neck pain (14.6%). The overall prevalence of asthma among adults is 7.6%, but this rate increased with obesity (11.1%), particularly among women (14.6%). In addition, a considerable number of women (26.7%) aged <45 years reported the regular use of oral contraceptives.

Given the large number of medications currently available, the number of research studies examining potential drug–nutrient interactions is quite limited. A Pubmed search revealed the number of published research studies describing the potential effects of drug–drug interactions exceeds those describing drug–nutrient interactions by 100 fold. Often the resources on drug–nutrient interactions that are readily available to health care providers simply repeat the same lists of outdated or obscure examples, and seldom provide any perspective on the degree or quality of the supporting evidence. A comprehensive, updated review of the potential drug–nutrient interactions with use of the most often prescribed medications for commonly diagnosed conditions among the general U.S. adult population is warranted. Thus, the goal of this review is to update the available evidence as it relates to these conditions, and enhance awareness of the potential nutrition-related problems with chronic use of commonly prescribed medications (Table 1).

## 2. Part I: Medications Most Likely to Affect Nutritional Status

### 2.1. Proton Pump Inhibitors (PPIs)

The main action of PPIs is to reduce gastric acid production. Thus, decreased absorption of micronutrients that depend on low pH for uptake into intestinal cells may occur with PPI use.

#### 2.1.1. Vitamin B12

Gastric acid is needed to remove B12 from dietary protein for intestinal absorption. The form of vitamin B12 in fortified foods and dietary supplements does not require gastric acid and proteolysis to liberate it from protein binding.

Conflicting evidence has been reported on the relationship between PPI use and vitamin B12 status in adults. Case–control and prospective cohort studies measuring serum B12 in older adults determined the use of PPIs for at least 12 months was associated with an increased risk of B12 deficiency [10,11,201]. This relationship persisted even when adjusting for multivitamin use or supplementation with B12 alone [10,11]. However, a cross-sectional analysis of elderly patients on PPI therapy >3 years found no significant difference in serum B12 levels compared to non-PPI users, after adjusting for age, C-reactive protein levels, and *H. pylori* infection [202]. It is possible the findings of this study reflect a “healthy user effect” given that PPI users who received parenteral vitamin B12 supplements, i.e., patients who developed a B12 deficiency as a result of PPI use, were excluded. Few long-term intervention studies have assessed the effects of PPI therapy on B12 status and the results of those studies are inconsistent [12,13]. Heterogeneity in study populations, e.g., age, adherence rate, small sample size, study design, dose, and the parameters chosen to assess B12 status (serum, urine, methylmalonic acid) may account for varied results among the cross-sectional and intervention studies [203]. 

Specific risk factors may make certain individuals more susceptible to a B12 deficiency with PPI use. Collective findings from small, short-term intervention studies indicate omeprazole impairs acid secretion and inhibits intestinal absorption of protein-bound B12, especially in smokers and during concurrent *H. pylori* infection, which augments the pH-increasing effect of PPIs and increases the risk for atrophic gastritis [13,14,15,16]. A prospective cohort study of 49 *H. pylori*-positive patients determined those who developed atrophic gastritis (~33%) while taking omeprazole for five years on average had significantly lower serum B12 levels than at baseline, while those who did not develop atrophic gastritis had no change in B12 status [12]. Age is another potential risk factor for B12 deficiency with PPI use. The previously described cross-sectional studies that showed a higher risk of B12 deficiency (as measured by serum B12 levels) with long-term PPI use were performed in adults >60 years [10,11]. Given that the elderly are already susceptible to B12 deficiency, this may be a nutrient–drug interaction of concern for this population [204]. Genetic polymorphisms that inhibit microsomal enzyme cytochrome P450, which metabolizes omeprazole, have also been shown to differentially affect serum B12 in patients. Specifically, those with a heterozygous mutation of this enzyme who metabolize omeprazole more slowly, had a higher intragastric pH and significantly lower levels of serum B12 after one year of omeprazole use than patients without the mutation [17,205].

Additionally, certain dietary choices may influence risk of deficiency. Evidence suggests the effects of omeprazole on B12 status are due solely to impaired gastric acid secretion and not impaired intrinsic factors [206]. Therefore, drinking acidic fruit juice concurrently with B12 may improve absorption in PPI users, as demonstrated in a small absorption study in hypochlorhydric older adults taking omeprazole [14].

In summary, PPI use has been observed to decrease protein-bound B12 absorption and may lead to B12 deficiency in some individuals, although the results are mixed. Reflecting this, the American Gastroenterological Association’s (AGA) 2017 clinical practice update concluded there is currently insufficient evidence to recommend routine screening of vitamin B12 status or routine supplementation of patients taking PPIs [207]. However, certain PPI users including the elderly, individuals with atrophic gastritis and/or *H. pylori* infection, and slow metabolizers of omeprazole may be at a higher risk of B12 deficiency.

#### 2.1.2. Vitamin C

Vitamin C is highly concentrated in gastric juice, where it is predominantly found in its biologically active antioxidant form, ascorbic acid (AA) [18]. In addition to acting as an antioxidant, AA in gastric juice functions to eliminate potentially carcinogenic nitrites from saliva [2,18]. In this process, AA is converted into its inactive form, dehydroascorbic acid (DHAA), which cannot be absorbed in the intestine. However, it may be converted back to AA through a pH-dependent process for reabsorption [18]. Treatment with 40 mg/d omeprazole for four weeks in volunteers with and without *H. pylori* infection significantly reduced the proportion of AA to total vitamin C concentrations in gastric juice from all volunteers and increased intragastric pH [18]. The interaction between omeprazole treatment and vitamin C was more pronounced in patients with *H. pylori* infection as these subjects also experienced significant reductions in total vitamin C concentrations in their gastric juice. This finding was later confirmed in another short-term omeprazole intervention study of similar design [19].

Observational and short-term intervention studies have demonstrated that PPI use may also be linked to the reduction of serum/plasma vitamin C levels in patients with *H. pylori* infection. In one observational study, patients with *H. pylori* infection had plasma vitamin C levels that were at least 30% less than *H. pylori*-negative patients [20]. However, the dietary intake of vitamin C in infected patients was also lower than in non-infected volunteers. Conversely, two short-term (four weeks) intervention studies found reduced circulating vitamin C levels in *H. pylori*-infected patients taking omeprazole independent of dietary intake [21,22].

Given the evidence that omeprazole increases the ratio of DHAA to total vitamin C in the gastrointestinal tract, lower circulating vitamin C levels with PPI use in *H. pylori*-infected patients may be due to decreased intestinal bioavailability of vitamin C. However, the exact mechanism underlying this observation has yet to be elucidated. To date, the clinical significance of the interaction between omeprazole and vitamin C is not clear.

#### 2.1.3. Iron

Non-heme iron is the predominant form of iron found in plant foods and must be reduced prior to absorption in the small intestine. Therefore, PPI use may affect its absorption. However, there is little conclusive evidence of an increased risk of iron deficiency in PPI users in the short term. A few case reports have documented that omeprazole-induced achlorhydria may impair the response to iron supplementation in patients who were previously iron-deficient [23]. No cases of impaired iron response with omeprazole use have been reported in iron-replete individuals. Furthermore, a small, exploratory, cross-over intervention study in nine healthy adults found no difference in iron absorption between the control phase and a four-day omeprazole treatment phase [208]. In a study of patients with Zollinger Ellison syndrome, a disease in which tumors cause the stomach to produce too much acid, PPI use was not associated with diminished iron status [209].

However, in a retrospective cohort study of adult patients, chronic PPI therapy for >1 year was associated with a significant decrease in hemoglobin, hematocrit, and mean corpuscular volume from baseline, while no hematological changes were observed in matched controls [210]. In a large case–control study of 77,046 patients diagnosed with iron deficiency, the odds ratio (OR) for developing this condition was 2.49 fold higher (95% confidence interval (CI): 2.35–2.64) for patients taking PPI for ≥2 years [211]. Additionally, PPI use for >1 year was found to decrease phlebotomy requirements for hereditary hemochromatosis patients. This same study also found that the use of PPIs for one week decreased the absorption of non-heme iron by 50% in these individuals [212].

In summary, there is some evidence to indicate PPI use may negatively impact iron absorption. PPI users already at elevated risk for iron-deficiency or those with pre-existing iron deficiency may be at greater risk for impaired iron absorption during PPI therapy. Similar to its stance on the routine monitoring of vitamin B12 status in those taking PPI, the AGA does not currently recommend routine monitoring of iron status [207].

#### 2.1.4. Calcium

As with the nutrients discussed above, calcium absorption in the small intestine is influenced by gastric pH. Therefore, similar concerns have been raised regarding PPI use, calcium absorption, and bone health in chronic PPI users [213]. A few observational studies have found an association between PPI use and an increased risk of fractures in individuals with other risk factors [24,25,26], while other studies reported a significant increase in subsequent anti-osteoporotic drug use after being prescribed PPIs, depending on duration [214]. Recent systematic reviews and meta-analyses of observational studies indicate there may be a modest increased risk of fracture with PPI use, but there is no evidence of a duration or dose–response effect, and the possibility of residual confounding cannot be ruled out [215,216]. Although a direct association between PPI use and fracture risk is possible, randomized controlled trials (RCTs) are needed to determine whether PPI use actually causes fractures.

Short-duration, controlled intervention studies that investigated the effect of PPI use on calcium absorption have also produced mixed results, i.e., some showed reduced absorption with PPI use [27,217] and others showed no difference [28,218]. Discrepancies in these findings may be due to calcium source (food vs. supplement), subjects’ age and whether they were in the fed or fasted state. To date, no long-term studies have investigated PPI use on calcium absorption. The effect of PPI use on bone mineral density (BMD) is also unclear as some studies have found either a modest inverse association [219,220] or no association [221].

In summary, the collective evidence indicates that chronic PPI use is associated with increased fracture risk, particularly in individuals who may already be at risk of fractures, such as older adults, and is considered a contributing factor to osteoporosis and fracture risk by the National Osteoporosis Foundation [222]. Despite this, the AGA does not yet recommend routine monitoring of BMD in PPI users [207]. Given the limited number of studies that have examined the effects of PPI therapy on calcium absorption and BMD, the mechanisms underlying their potential effects are not fully understood.

#### 2.1.5. Magnesium

Case reports of hypomagnesemia with chronic PPI use have been widely documented [29,30]. In some, but not all cases, magnesium supplementation alone was not completely successful in reversing hypomagnesemia until PPI therapy was discontinued [31]. Among ~30 cases of hypomagnesemia, more than half of these patients received PPI therapy for ≥5 years and 30% for ≥10 years [223]. All of these reported cases were in older adults (age 51–82 years) and occurred more frequently in women than men [223]. 

The mechanism underlying hypomagnesemia secondary to PPI use is unknown, and no well-designed studies to accurately determine the effect of PPIs on magnesium status have been conducted [29].

#### 2.1.6. Zinc

Gastric acid secretion may play a role in intestinal zinc absorption in humans, reflecting the need for dietary zinc to be in the reduced state [224]. In one study, omeprazole administration at 60 mg/d for one week in healthy adults almost doubled the pH of fasting gastric juice and decreased plasma zinc levels by ~40%. These results suggest omeprazole may decrease zinc absorption by increasing gastric pH, however there was no control group and the sample size was small [225]. A more recent study found that in healthy control subjects the plasma zinc increased 126% in response to supplementation with 26.2 mg zinc twice daily for 14 days, compared with only a 37% increase in those on long-term PPI therapy [226]. In this study, baseline plasma zinc levels were also negatively associated with long-term PPI use.

Long-term studies are needed to test the effects of chronic PPI use on zinc status. To date, the clinical implications regarding the decreased absorption of zinc with PPI therapy are unclear.

#### 2.1.7. Beta (β)-Carotene

Thus far, only one study has investigated the effect of PPI therapy on the absorption of the provitamin A carotenoid, β-carotene [227]. In a crossover study of 12 healthy volunteers, plasma β-carotene levels at 6 and 24 h after supplementation with 120 mg were significantly lower following seven-day treatment with 40 mg/d omeprazole compared with no omeprazole treatment.

Very limited research indicates PPI use may inhibit the absorption of this carotenoid, and the potential clinical implications are unclear.

### 2.2. NSAIDs: Aspirin

#### 2.2.1. Vitamin C

Studies conducted in the 1970s were among the first to discover that high doses of aspirin for the treatment of arthritis may negatively impact vitamin C levels in patients. In one study plasma ascorbic acid levels were found to be abnormally low in patients with rheumatoid arthritis (RA) compared to healthy controls, but only RA patients taking high doses of aspirin had significantly lower platelet ascorbic acid, a better measure of tissue vitamin C levels, when compared to controls [228]. A later study tested the effect of 500 mg vitamin C with and without 900 mg aspirin on plasma, leukocyte, and urinary ascorbic acid in young, healthy adults [32]. Although all measures of vitamin C were higher following supplementation, the observed increase was significantly lower among those who were also given aspirin.

Based on studies that found increased fecal vitamin C in guinea pigs given vitamin C and aspirin compared to vitamin C alone, investigators hypothesized aspirin may inhibit the intestinal absorption of this vitamin [32]. However, a small intervention study conducted in six healthy men found that while 600 mg aspirin completely inhibited the uptake of ascorbic acid in leukocytes over 2 h, regardless of supplemental vitamin C dose (500–2000 mg), the plasma vitamin C response was not affected by aspirin [33]. Investigators from this study concluded that aspirin may not inhibit gastrointestinal absorption, but may impact the storage of vitamin C in leukocytes by inhibiting its uptake into these cells. Interestingly, experiments on the effect of this interaction in subjects with the common cold indicate that, unlike healthy individuals, increases in leukocyte vitamin C concentrations did not differ when 2000 mg vitamin C was given with or without 600 mg aspirin, suggesting the cold virus may modify the interaction [34].

A more recent prospective, randomized, double-blind, parallel-arm study in healthy men and women found 2400 mg aspirin for six days reduced vitamin C concentrations in urine, plasma, and particularly gastric mucosa [35]. Decreased vitamin C in gastric mucosa may be due to increased antioxidant defenses in response to aspirin-induced mucosal damage, rather than impaired intestinal absorption [35]. This hypothesis is supported by several in vivo and in vitro studies in which the co-administration of vitamin C and aspirin decreased the number of aspirin-induced gastric lesions and increased gastric tolerability [229,230,231,232].

In summary, acute and short-term doses of aspirin taken concurrently with vitamin C may alter vitamin C absorption in leukocytes, and its antioxidant activity in gastric mucosa may protect mucosal cells from damage. However, the effects of long-term, low-dose aspirin on vitamin C status, and its clinical implications, are unclear. Given the evidence that vitamin C may help prevent aspirin-induced gastric lesions, patients suffering from gastric mucosal injury due to an aspirin regimen may benefit from vitamin C supplementation [233].

#### 2.2.2. Iron

It is well established that aspirin use can cause gastric mucosal damage, gastric ulcers, and increase the risk of gastrointestinal bleeding, even at low doses [234,235,236,237]. Therefore, it is possible a long-term aspirin regimen may decrease iron stores, increasing the risk for iron-deficiency anemia. A retrospective study of elderly patients (mean age 82 years) with diagnosed iron deficiency anemia found the prevalence of aspirin treatment among those with anemia (24%) was more than double that of the general primary care population (11%), suggesting that regular aspirin use may be a contributing factor in this age group [36]. Conversely, a more recent study found no association between the prophylactic use of aspirin and the prevalence of anemia among adults aged 18–85 years, only 40% of whom were >60 years [238].

Several studies have assessed the relationship between aspirin therapy and hemoglobin (Hb) levels, mostly in older adults, as this group is commonly prescribed a prophylactic aspirin regimen. One retrospective study in a primary care population (mean age 66 years) found mean Hb levels declined significantly in men taking low-dose aspirin, but not in women [239]. A controlled trial in which subjects ≥70 years were randomized to take 100 mg/d aspirin or placebo for one year, found the aspirin-treated subjects had a significant decrease in mean Hb levels compared to the control group [240]. However, in both studies, Hb levels remained in the normal range and thus did not indicate the presence of iron deficiency anemia.

The authors of a recent narrative review analyzed the evidence supporting low dose aspirin as a cause of anemia, particularly in the absence of overt bleeding, but found significant limitations in the available evidence and considerable heterogeneity between studies [37]. Collectively, the associations between aspirin, anemia, and Hb were inconsistent, and it is unclear whether low-dose aspirin causes iron deficiency anemia. However, among older adults the association between low dose aspirin use and decreased Hb was notable.

One limitation to using Hb as a marker of iron status is that it is generally considered to be the last parameter to change. That is, early stages of iron deficiency occur well before measurable changes in Hb are observed. Two separate studies, the first conducted in a population of Danish males, aged 40–70 years [241], and the second in a population of U.S. adults, mean age 76 years [38], reported an inverse association between aspirin use and serum ferritin concentrations, an indicator of iron stores. Although the first study did not adjust for additional factors that may influence serum ferritin and included only men taking low (150–325 mg/d) and high (1–3 g/d) doses, the second adjusted for a large number of confounding factors and analyzed both men and women on a low-dose (325 mg/d) regimen. Another study determined the combination of *H. pylori* infection and aspirin use was associated with significantly lower serum ferritin in women, but not men [39]. Given that serum ferritin is a well-known inflammatory marker, these studies cannot rule out the possibility that decreased serum ferritin from aspirin use is a result of the anti-inflammatory effects of this drug, rather than a result of depleted iron stores.

In summary, due to limitations in the measures of iron status and heterogeneity between studies, the effect of aspirin use on iron status is not completely understood. However, older adults on a low-dose regimen, particularly those with *H. pylori* infection, may be at risk of decreased Hb and serum ferritin.

### 2.3. Anti-Hypertensives: Diuretics

#### 2.3.1. Calcium and Loop Diuretics

Evidence in animal models indicates loop diuretics enhance calcium excretion and decrease calcium status, while thiazide diuretics inhibit calcium excretion and may enhance calcium status [242,243]. In vitro and in vivo studies in perfused animals showed that loop diuretics (especially furosemide) inhibit calcium reabsorption in the thick ascending loop of Henle by inhibiting the lumen-positive trans epithelial voltage that drives passive calcium-ion transport in this part of the kidney [243,244,245]. 

In healthy adults, both single and short-term doses of 80 mg furosemide have been shown to increase calcium excretion and plasma parathyroid hormone (PTH) [40,41,246]. Furthermore, a crossover, placebo-controlled RCT in 20 postmenopausal osteopenic women demonstrated that the effect of loop diuretics (0.5–2.0 mg/d bumetanide) on urinary calcium and plasma PTH is dose-dependent [42]. Collectively, these findings indicate that loop diuretics negatively affect calcium homeostasis, which may lead to secondary hyperparathyroidism.

Studies examining the effect of loop diuretics on BMD have also been conducted. An observational study in healthy elderly women reported significantly lower hip BMD in loop diuretic users, after adjusting for age, years since menopause, and body weight [43]. Conversely, a case–control study in postmenopausal women observed no difference in BMD between long-term (≥2 years) loop diuretic users vs. nonusers, despite increased urinary calcium and plasma PTH in users [44]. The authors of this study concluded that, because loop diuretic users also had higher 1,25-dihydroxyvitamin D levels compared with nonusers, renal calcium losses were compensated with increased intestinal calcium absorption; thus the calcium balance remained neutral and no major effects on bone metabolism were observed.

In a double-blind RCT in osteopenic postmenopausal women, treatment with bumetanide for one year significantly decreased BMD by 2% and increased markers of bone turnover compared to a placebo [45]. An annual BMD loss of 1–2% may have consequences on fracture risk if accumulated through several years of treatment. All participants in this study were supplemented with 800 mg/d calcium and 10 µg/d vitamin D throughout the intervention period. The researchers concluded that bumetanide may antagonize the potential beneficial effect of calcium and vitamin D supplementation on bone health, although the absence of a non-supplemented group did not allow for a definitive conclusion in subjects with low calcium intake.

Several observational studies have examined the association between loop diuretic use and fracture risk in elderly and middle-aged adults [46,47,48]. In one case control study of elderly patients hospitalized for a hip fracture vs. age- and sex-matched controls, the risk of hip fracture was 3.9 times greater for current users of furosemide compared to nonusers after adjusting for several confounding variables, including the use of other diuretics [46]. Similarly, in a cohort study of elderly women >70 years, loop diuretic use for five years was associated with an increased risk of any osteoporotic fracture after adjusting for confounders [48]. In a younger cohort of middle-aged adults, loop diuretic use during the previous five years was associated with a 4% increased risk of any fracture and a 16% increased risk of hip fracture after adjustment [47]. In this study the use of furosemide was associated with a higher risk than bumetanide. The authors also observed a tendency towards decreased fracture risk with increased dose among current users, but in former users this risk increased with dose.

In summary, loop diuretics increase urinary calcium excretion, although calcium balance may possibly be maintained by increased intestinal calcium absorption. Despite this potential compensatory mechanism, some individuals, particularly older adults and the elderly who absorb intestinal calcium less efficiently with age, may be at higher risk of decreased BMD and increased fracture risk with chronic use. Other risk factors for this association include the dose, duration, and form of loop diuretic. 

#### 2.3.2. Calcium and Thiazides

Thiazides primarily influence the early distal tubule diluting site of the kidney [243] and, thus, have a greater effect on osmotic vs. water diuresis, leading to increased calcium reabsorption [247,248,249,250]. Daily administration of 200 mg hydrochlorothiazide for four days has been shown to decrease urinary calcium in both healthy individuals and subjects with hyperparathyroidism, but not in those with hypoparathyroidism [49]. This finding suggests that, in addition to direct effects in the kidney, thiazides may also impact urinary calcium excretion through a PTH-dependent mechanism. A crossover RCT in postmenopausal osteopenic women demonstrated that decreases in urinary calcium with thiazide treatment at 2.5–10 mg/d are dose-dependent [42]. Although no change in PTH was observed in this study, thiazide treatment increased plasma osteocalcin, a marker of bone formation. Similarly, a small pilot study in men fed low calcium diets found hydrochlorothiazide inhibited bone resorption, regardless of whether or not vitamin D was co-administered with treatment [251]. Despite these findings, an investigation into the determinants of BMD in healthy older women found thiazides did not significantly influence BMD at any site [43]. 

To date, no RCTs have examined the effect of thiazide diuretics on BMD and fracture risk. However, the data from a number of observational studies have been examined in two different meta-analyses [50,51]. Both analyses reported a reduced risk of hip fracture (18–24%) with thiazide use, and one found long-term use is more protective against fracture risk than short-term therapy. Both reports concluded that RCTs are needed before definitive recommendations can be made regarding the use of thiazides to protect against fractures.

A population-based descriptive study investigating the incidence of hypercalcemia in thiazide users reported an age- and sex-adjusted incidence of 7.7 per 100,000 person-years in a predominantly Caucasian population, with an even higher rate of 55.3 among elderly women, age 70–79 years [52]. In this study, the most typical form of hypercalcemia was mild, uncomplicated, non-progressive, and discovered approximately six years after initiation. In contrast, a post hoc analysis of a RCT conducted in a population of healthy black adults who were taking either placebo or vitamin D at 1000–4000 IU/d found that, although hydrochlorothiazide users had higher serum calcium levels than nonusers, only one user had serum calcium levels high enough to be classified as hypercalcemic after three months on vitamin D [53]. The authors of this study did not quantify the length of time subjects were taking thiazides prior to the start of supplementation. Investigations into the effect of thiazides on hypercalcemia are limited, but it appears the frequency is fairly low among users, even when taking vitamin D supplements, and that older, Caucasian women on chronic thiazide diuretic therapy are the most susceptible. 

In summary, chronic thiazide use leads to reabsorption of renal calcium and may increase serum calcium levels above the normal range in certain individuals, particularly older women. Although observational studies indicate thiazide diuretics may protect against hip fractures, RCTs are needed to confirm these findings.

#### 2.3.3. Magnesium

Extensive evidence indicates that mild magnesium depletion is relatively common with loop and thiazide diuretic use. Loop diuretics directly inhibit magnesium reabsorption in the kidney, thus, both short and long-term treatment can lead to depletion [252]. Conversely, thiazides induce magnesium excretion indirectly through multiple mechanisms, including suppression of PTH [253]. Therefore, long-term therapy with thiazides is more likely to cause magnesium depletion than acute thiazide exposure [254].

Cross-sectional studies in patients with uncomplicated hypertension, arterial hypertension, or congestive heart failure suggest that chronic diuretic therapy modestly decreases serum magnesium concentrations but significantly depletes cellular magnesium concentrations, potentially leading to hypomagnesaemia [70,255]. Population studies that investigated risk factors for hypomagnesaemia with diuretic use have determined patients with congestive heart failure who receive high doses of loop diuretics on a chronic basis, elderly patients, and individuals with poor dietary magnesium intake, or high alcohol intake may be at increased risk [55,56,57]. 

Oral magnesium supplementation has been shown to be effective in increasing muscle concentrations of magnesium. In patients with arterial hypertension or congestive heart failure, magnesium concentrations were restored to normal after six months of supplementation [256].

#### 2.3.4. Thiamin

Evidence from both animal and human studies has demonstrated that acute doses of loop diuretics increase urinary loss of thiamin [257,258]. In these studies thiamin excretion rates were correlated with urine flow rate, indicating the loss of thiamin was due to increased and sustained diuresis, and was not specific to one particular diuretic [258]. Studies investigating thiamin status in patients with congestive heart failure taking any loop diuretic [58], or specifically furosemide [59,60], have consistently shown the prevalence of biochemical thiamin deficiency is significantly higher compared to age-matched controls. Furthermore, the prevalence of biochemical thiamin deficiency increased with an increasing dose of furosemide [59,60].

The effect of diuretics on thiamin is of particular concern for the elderly, who are at an increased risk of thiamin deficiency due to low dietary intake of this vitamin [259]. A prospective study focused exclusively on older adults (mean age 70 years) found decreased thiamin status during hospital stays correlated significantly with cumulative dosage of furosemide, adjusted for duration, indicating that this population is at higher risk of thiamin deficiency [61]. However, the increased risk of deficiency is not limited to hospitalized patients. An examination of the relationship between diuretic therapy and dietary intake of thiamin in 324 homebound older adults, >60 years, from the North Carolina Nutrition and Function Study, indicated that diuretic users were 2.3 and 4.2 times more likely to have intakes below the recommended dietary allowance and estimated average requirement for thiamin, respectively, compared to nonusers, even after adjusting for sociodemographic and meal pattern variables [62].

#### 2.3.5. Zinc

Several small human studies have demonstrated that treatment with a thiazide diuretic increases urinary excretion of zinc in patients with hypertension [63,64,65,66], but not in healthy adults [260]. The results of follow-up studies that measured serum zinc in patients taking diuretics were mixed. A pilot study of hypertensive patients taking either 50 mg/d hydrochlorothiazide plus 5 mg/d amiloride, 25 mg/d hydrochlorothiazide alone, or healthy controls, showed no difference in serum zinc between the three groups despite the increased excretion of urinary zinc in both diuretic groups vs. healthy controls [66]. At 10–14 mg/d, the dietary intake of zinc in this population was adequate. Conversely, a small study of hypertensive, middle-aged men found hydrochlorothiazide treatment at 25–50 mg/d for at least six months was associated with lower serum zinc levels compared to healthy, un-medicated, age-matched controls [67]. In a subgroup of individuals from this study who were taking diuretics, supplementation with 500 mg zinc for 30 days significantly increased serum zinc levels to within the normal range (11.6–19.1 µmol/L), comparable to levels observed in the healthy control subjects. In a study that measured both serum and hair zinc levels, hypertensive patients treated with thiazide for 6–36 months had lower mean hair, but not serum, zinc compared to mildly hypertensive patients who were not treated with diuretics for six months prior [261]. In addition, post-mortem zinc levels measured in liver and skeletal muscle were reported to be significantly lower in those who used diuretics for more than six months prior to death compared to nonusers [68].

Urinary zinc depletion with thiazide diuretic use may lead to tissue depletion, although it is unclear to what extent diuretics alone lead to clinical zinc deficiency. It is also possible the loss of zinc from thiazide diuretic use may be additive to other risk factors for zinc deficiency, such as inadequate intake, hepatic cirrhosis, diabetes mellitus, gastrointestinal disorders, or renal disease [63,64,69].

#### 2.3.6. Potassium

Although both loop and thiazide diuretics increase urinary potassium excretion with chronic use, most research has focused on the latter since they generate hypokalemia more frequently than the former [262,263,264]. 

Unlike loop diuretics, which directly inhibit potassium reabsorption in the loop of Henle, thiazide use stimulates renal potassium secretion via multiple mechanisms [263,264]. A computerized search of thiazide prescriptions and electrolyte abnormalities in six United Kingdom general practices found that, of 951 patients whose electrolyte levels were measured while on thiazides, hypokalemia occurred in 8.5% and was positively associated with dose [71]. A meta-analysis that investigated the dose–response relationships between serum potassium and use of hydrochlorothiazide, chlorthalidone and bendroflumethiazide, found bendroflumethiazide to be the most potent at reducing potassium, while hydrochlorothiazide was the least potent [72]. This finding confirmed a previous meta-analysis that compared doses of only hydrochlorothiazide and chlorthalidone on serum potassium [73]. Thiazide-induced hypokalemia has been shown to be associated with increased blood glucose [262,265] and ventricular arrhythmias [74,263], although evidence on the latter has been mixed [266,267]. 

Despite concurrent potassium supplementation with 31 mmol/d potassium and chronic diuretic use for 2–14 years, muscle concentrations of potassium were found to be significantly lower in diuretic users than age-matched controls [70]. A similar observation was reported in a double-blind RCT in hypertensive men [74]. In this study, serum potassium decreased with thiazide use compared to placebo, but the concurrent use of potassium supplements prevented hypokalemia and ventricular arrhythmias. While potassium supplements may not fully restore serum or body tissue potassium levels to normal, they appear to be effective in preventing hypokalemia. 

In summary, potassium supplements can be used in individuals presenting with symptoms of hypokalemia due to chronic loop or thiazide diuretic use [268]. However, potassium supplementation may further decrease blood pressure in addition to the effects of the diuretic itself, potentially resulting in low blood pressure [269]. An alternative strategy may be to use potassium-sparing diuretics, which not only help prevent the loss of potassium but also other ions, such as magnesium, that may similarly be reduced with loop and thiazide diuretic use [268]. 

#### 2.3.7. Folate

Two case reports dating back to 1968 documented low serum folate levels and megaloblastic anemia in patients taking the potassium-sparing diuretic, triamterene, in addition to hydrochlorothiazide [75]. Both patients had alcoholic cirrhosis. Administration of folic acid to one patient who became critically ill due to this condition showed significant improvement. Structurally, triamterene looks similar to folic acid, and in vitro studies indicate high doses of this diuretic inhibit dihydrofolate reductase in leukocytes, an enzyme essential in folate metabolism for the synthesis of nucleotides [270]. Concentrations of triamterene required to inhibit this enzyme and cause megaloblastic anemia may occur in individuals with impaired liver function, such as alcoholic cirrhosis. However, healthy adults who quickly metabolize this drug are unlikely to be at risk. This was confirmed in a cohort study from 1991 in which free-living subjects on chronic triamterene therapy were reported to have similar serum folate, hemoglobin and red blood cell counts as nonusers [76]. By contrast, in an observational study conducted after the implementation of folate fortification (1998), long-term use of diuretics was associated with lower red blood cell folate in hypertensive patients [271]. Additionally, a short trial of hypertensive patients found decreased folate levels with hydrochlorothiazide use after six weeks [272]. These results suggest that diuretics other than triamterene, namely thiazides, may negatively influence folate status, even in the age of folate fortification. However, the clinical significance of this drug–nutrient association is unknown.

### 2.4. Anti-Hypertensives: Angiotensin-Converting Enzyme (ACE) Inhibitors

#### 2.4.1. Zinc

Long-term treatment with ACE inhibitors, particularly captopril, can cause hypogeusia. Given that the loss of taste is a symptom of zinc deficiency, the effects of captopril on taste acuity and zinc status were investigated in hypertensive patients on long-term (>6 months), high-dose (266 mg/d) captopril treatment, short-term (<6 months), lower-dose (100 mg/d) treatment, and controls not taking captopril. Patients in the long-term, high-dose group were reported to have higher taste detection and recognition thresholds, lower plasma zinc levels, and higher urinary zinc excretion compared to controls, suggesting the loss of taste in response to captopril may be associated with decreased zinc status. No significant differences were found between the short-term, lower-dose captopril group and controls. Similarly another captopril intervention study in hypertensive patients found no change in serum zinc after 5–6 months on 100 mg/d captopril, indicating the effect of captopril on zinc status may be dose-dependent [77]. However, lower doses (e.g., 50 mg/d) were found to lower zinc status in patients presenting with either kidney disease or heart failure [69,78,79]. Collectively, these findings indicate captopril may lower zinc status and increase the risk of zinc deficiency, but only with chronic use at higher doses, or in individuals with hypertension and comorbidities.

Specific ACE inhibitors may affect the occurrence or severity of zinc depletion differently. Two separate studies that compared the effects of captopril and enalapril on markers of zinc status found that, although both ACE inhibitors decreased zinc status compared to untreated hypertensive patients or healthy controls, chronic captopril use had a larger effect on zinc status than enalapril [80,81]. In a different randomized, double-blind study, both captopril and benazepril lowered serum zinc and increased urinary zinc in patients with essential hypertension after four weeks [82]. However, after eight weeks, serum zinc decreased more significantly with captopril use.

In summary, ACE inhibitors, as a drug class, may increase the risk of zinc deficiency. However, this effect is more pronounced with captopril than other ACE inhibitors. The underlying mechanism may be due to the thiol-radical group present in captopril that can chelate serum zinc and enhance its excretion [2]. A major limitation of all these studies is that serum and plasma zinc levels do not always correlate with tissue levels of this mineral [273]. Studies measuring a more comprehensive array of zinc levels in the human body are needed to ascertain whether these drugs alter zinc distribution in tissues or zinc function. Nevertheless, the evidence to date suggests patients on chronic ACE inhibitor treatment, especially captopril, may be at higher risk of impaired zinc status, particularly when other factors associated with impaired zinc status—such as heart failure, renal disease, older age, malabsorption and diarrhea—are present [83].

#### 2.4.2. Potassium

ACE inhibitors can cause retention of potassium in the kidney via their inhibitory effect on aldosterone secretion [274,275]. Although the incidence of hyperkalemia associated with ACE inhibitor use in hypertensive patients is 1–2% [86], two case reports documented older adults consuming high potassium, sodium-restricted diets may be at greater risk of developing hyperkalemia with ACE inhibitor use [87,88]. Additionally, a small retrospective analysis of patients on ACE inhibitors reported the incidence of hyperkalemia to be higher in those with chronic renal failure and diabetes [89]. Others reported the risk of hyperkalemia to be greater in ACE inhibitor patients who have congestive heart failure [86,90], especially if they also consume potassium supplements or potassium-rich foods [86,91]. In a placebo-controlled trial, subjects taking enalapril for 40 months had a three-fold higher rate of hyperkalemia than placebo [90]. Collectively, these findings indicate that certain factors may contribute to increased risk of hyperkalemia with ACE inhibitor use, including older age, renal disease, diabetes, congestive heart failure, use of potassium-sparing diuretics, potassium supplements or consumption of potassium-rich diets.

### 2.5. Anti-Hypertensives: Calcium Channel Blockers (CCBs)

#### 2.5.1. Folate

Gingival hyperplasia may develop in response to CCB treatment [92,93,94,95,96], mostly in men, but also in women [97]. Although likely a drug class effect, the largest number of case reports have been documented with the use of nifedipine [98]. A case–control study from The Netherlands showed current use of CCBs doubled the risk of gingival hyperplasia in a dose-dependent manner [99]. The association between CCB use and gingival hyperplasia has been confirmed in other studies that investigated the incidence of this condition in users of amlodipine [276] and nifedipine [277] when compared with controls.

In addition to the presence of dental plaque and poor oral hygiene, a major contributing factor to the development of gingival hyperplasia is impaired uptake of folate into gingival fibroblasts [97,278]. Folic acid supplementation has been shown to decrease the incidence, reduce the severity, or delay the onset of gingival hyperplasia [279,280,281,282]. However, these studies were conducted in epileptic children and adults who developed gingival hyperplasia due to anti-seizure medications. RCTs examining CCB-induced gingival hyperplasia in older adults are absent. Meanwhile, patients taking CCBs should be counseled on how to meet their recommended folate intake.

#### 2.5.2. Potassium

Two case reports of hyperkalemia/hypotension in older adults taking CCBs initiated discussion of the effect these drugs may have on potassium status in the elderly [100,101]. In both cases it should be noted patients were also taking a beta blocker. Conversely, two retrospective cohort studies in hypertensive patients who were taking either a CCB or an alternative anti-hypertensive medication found no association between CCB use and increased serum potassium [102,103].

In summary, very limited data indicates that CCB monotherapy does not appear to influence potassium status, while the concomitant use of beta-blockers may be of concern in some older adults.

### 2.6. Hypercholesterolemics: Statins

#### 2.6.1. Coenzyme Q10 (CoQ10)

CoQ10 is a naturally occurring, fat-soluble, vitamin-like compound obtained from the diet and, to a lesser extent, from endogenous synthesis. CoQ10 functions in the electron transport chain in the mitochondria and, thus, plays an important role in energy metabolism. CoQ10 is an intermediate in the mevalonate pathway, which is inhibited by statins [283].

A number of studies have consistently reported that statin treatment lowers serum CoQ10 levels in hypercholesterolemic patients, particular the elderly [104,105,106,284,285,286,287,288,289,290,291,292]. Some of these studies have further shown that this effect is dose-dependent [104,105,106], and supplementation with CoQ10 effectively increases the blood levels of this compound in statin patients [286,289]. However, changes in serum CoQ10 do not necessarily reflect intramuscular changes [107,293]. Given its role in mitochondrial function, the amount of CoQ10 in muscle is likely a more clinically relevant measure than circulating concentrations. 

Studies investigating the effect of statins on intramuscular CoQ10 are limited and inconsistent [107,108,109,294]. The one study that reported decreased intramuscular CoQ10 levels with statin use differed from the others in that both men and women were evaluated (as opposed to men only), subjects >55 years were included, and either the dose of statin given was higher (80 vs. 20 mg) or the treatment duration longer (eight vs. two weeks). All of the studies were limited in size.

While the effect of statins on intramuscular CoQ10 levels are unclear, levels were reported to be lower in individuals with statin-associated myopathy and mitochondrial myopathy [295,296]. It has yet to be determined whether depletion of intramuscular CoQ10 causes statin-associated myopathy or if it is a byproduct of this condition [297,298,299]. Intervention studies investigating the effect of CoQ10 supplementation in statin-associated myopathy patients have produced mixed results. In cancer patients receiving extremely high statin doses (240 mg/d), CoQ10 supplementation decreased the severity of myopathy symptoms, but not the frequency of this condition [300,301]. Randomized trials conducted in older men and women with statin-induced myalgia who were supplemented with CoQ10 at either 100 mg/d for 30 days [110,111] or 200 mg/d for 90 days [112] reported reduced statin-related muscle pain and other symptoms of myopathy compared with controls. Conversely, two other trials of similar design conducted in older adults supplemented with 120–200 mg/d for 90 days found no effect on statin-associated myalgia [113,114]. However, the latter studies used a different outcome measure for determining myalgia symptoms compared to the former studies, i.e., the Visual Analogue Scale vs. Brief Pain Inventory. Given the mixed findings, well-designed RCTs to elucidate the efficacy of CoQ10 on statin-induced myopathy are needed.

In summary, statin use may lower serum CoQ10 levels in a dose-dependent manner but the clinical implications are unclear, and it is unknown whether decreases in serum CoQ10 result in significant depletions in muscle. The risk of CoQ10 depletion in muscle may be greater in older adults, as well as patients who develop myopathy while taking statins. The efficacy of CoQ10 supplementation on statin-induced myopathy symptoms is still under debate.

#### 2.6.2. Vitamin D

A number of studies have investigated the relationship between statin use and vitamin D status as measured by circulating 25-hydroxyvitamin D and 1,25-dihydroxyvitamin D, but the findings from these studies are quite controversial. A prospective cohort study of 91 hyperlipidemic patients, 17 of whom were diabetic and 43 with systemic hypertension, who had not previously been treated with statins, were given rosuvastatin for eight weeks [115]. Compared with baseline levels, statistically significant increases in 25-hydroxyvitamin D (14.0 to 36.3 ng/mL) and 1,25-hydroxyvitamin D (22.9 to 26.6 pg/dL) were observed. The same authors later reported that 25-hydroxyvitamin D levels increased with rosuvastatin when compared with fluvastatin treatment in a trial of hyperlipidemic patients [116]. Their findings generated a great deal of controversy based on the lack of a biologically plausible mechanism, the absence of control groups, and the apparent lack of adjustment for confounding factors such as vitamin D supplementation, physical activity, dietary vitamin D intake, clothing habits, and UV light exposure, i.e., the location of the study was conducted at higher altitudes where vitamin D synthesis can still occur during winter months [302,303]. Additional concerns regarding the potential interference of rosuvastatin and its metabolites on the antibody assay used to measure vitamin D have also been reported [302,304]. Nevertheless, there is consensus that a large, well-designed multicenter trial should be conducted to determine the effect of rosuvastatin on vitamin D status [303,305].

Subsequent investigations into the effect of statins on vitamin D status are mixed. A prospective open-label study in non-diabetic dyslipidemic patients assigned to 20 mg/d atorvastatin or 10 mg/d rosuvastatin for 12 weeks did not find any significant change in vitamin D status with either statin [117]. Similarly, a longer duration (one year), placebo-controlled trial in postmenopausal women found no effect of simvastatin at a higher dose (40 mg/d) on vitamin D status [118], and studies assessing the effect of pravastatin on vitamin D and its metabolites found no change with treatment after eight weeks [119,120]. There were several limitations to these studies, including sample size, lack of control group, residual confounding from season and sunlight exposure on vitamin D status, significant heterogeneity in study populations, statin treatment (specific drug and duration), and methods for measuring vitamin D, all of which are possible explanations for the inconsistent results. Interestingly, studies in which vitamin-D-insufficient individuals or patients with acute ischemic heart disease were treated with either rosuvastatin or atorvastatin have consistently shown increased vitamin D levels with treatment [121,122,123,124].

Other study findings that further complicate what is known about the relationship between statins and vitamin D include observations that there is a higher prevalence of suboptimal 25-hydroxyvitamin D levels in patients experiencing myopathic symptoms from statin use compared to those who do not [2,125]. Furthermore, supplementation with vitamin D regressed myopathic symptoms in >90% of patients. Some studies have also shown that patients who stopped statin treatment due to the development of myopathy were able to resume treatment without symptoms when taken in conjunction with vitamin D supplements [126,127], although one retrospective cohort study contradicted these findings [128].

In summary, the relationship between statins and vitamin D status remains controversial, and it appears that the nature of this particular drug–nutrient interaction is complex. Some studies indicate that some hyperlipidemic or vitamin-D-deficient individuals may improve their vitamin D status while taking statins. Conversely, vitamin D levels may be lower in patients with statin-induced myopathy.

#### 2.6.3. Vitamin E and β-Carotene

Vitamin E and β-carotene are transported in the circulation, in part, by low density lipoprotein (LDL) cholesterol, and a few studies investigating whether statins influence the status of these nutrients have been conducted. One retrospective analysis of patients with metabolic syndrome reported that statin-treated patients had significantly higher levels of vitamin E compared to their non-treated counterparts [306]. This differs from the findings of a three-month, placebo-controlled trial of normocholesterolemic patients with type 2 diabetes (T2D), in which 10 mg atorvastatin decreased unadjusted plasma vitamin E, and did not change the vitamin E–LDL ratio [307].

In a double-blind RCT of hypercholesterolemic Swedish men, treatment with 40 mg/d simvastatin for six weeks significantly reduced both total and LDL cholesterol and, consequently, circulating β-carotene levels [308]. However, when β-carotene levels were adjusted for cholesterol, concentrations increased significantly with simvastatin treatment. Similarly, two intervention studies that examined the effect of 12–14-week simvastatin treatment on circulating vitamin E levels concluded that the observed reductions in vitamin E were likely a function of decreased total and LDL cholesterol [287,290]. In a longer-term, open-label uncontrolled Finnish study of simvastatin and atorvastatin, short-term reductions in serum vitamin E and β-carotene concentrations (unadjusted for lipids) were reported after 12 weeks [309]. At 52 weeks, unadjusted serum β-carotene concentrations returned to baseline, but vitamin E remained reduced for both statin treatments. Similar to the Swedish study, the ratios of each nutrient to LDL cholesterol were significantly elevated for both statins at 12 and 52 weeks [309]. Another open-label, uncontrolled study of similar design, but shorter duration, showed simvastatin treatment for eight weeks had no effect on β-carotene concentrations, but lipid-corrected concentrations of vitamin E increased significantly from baseline [310].

In summary, statins are very commonly prescribed and may be associated with changes in circulating β-carotene and vitamin E levels. However, the effect of statins on these compounds is unclear. Additional, high-quality, controlled studies are needed to better understand potential interactions.

### 2.7. Oral Hypoglycemics: Metformin

#### Vitamin B12 

Cross-sectional analyses of adult populations with T2D from the U.S., Korea, the Netherlands and Brazil have consistently reported lower serum or plasma B12 in those taking metformin when compared to either healthy controls or T2D patients not taking metformin, after adjusting for covariates [129,130,131,132,133,134,135,311,312,313]. Compared to metformin and insulin treatment, the combination of metformin and sulfonylurea was associated with lower serum B12 and a higher prevalence of B12 deficiency [314]. Furthermore, in most of these studies the association between circulating B12 and metformin use was duration- and dose-dependent [129,130,131,132,133,134,135]. However, the prevalence of B12 deficiency among metformin users spanned a wide range (6–28%), due to differences in the defined level of deficiency between studies. 

Measuring both circulating serum vitamin B12 levels and a functional biomarker, such as methylmalonic acid (MMA), is preferred over either alone [315], yet only a few studies included such complementary biomarkers of B12 status. Cross-sectional and prospective studies that measured MMA and homocysteine (Hcy), in addition to serum B12, reported elevated MMA and Hcy, as well as lower serum B12 in T2D patients [136,316]. In another study, no difference in MMA was observed in T2D patients exposed to metformin for >1 year when compared to nonusers, though serum B12 was lower and Hcy slightly higher in the metformin group [317]. One additional study found that Hcy levels did not differ significantly between T2D patients using metformin compared to controls, despite a difference in serum B12 levels between groups, but Hcy levels were positively correlated with dose and duration of treatment [318].

A 16-week RCT that tested the effect of metformin treatment on serum Hcy and vitamin B12 in T2D patients found that it increased Hcy by 4% and lowered serum B12 by 14% with no changes observed in the placebo group [319]. Serum folate levels also decreased by 7% with metformin use, raising the concern that elevated Hcy in response to metformin may be attributable to decreased folate status, and not solely to decreased vitamin B12 status. Similar observations were made in another RCT investigating the long-term effect (~4 years) of 850 mg/d metformin on vitamin B12 and folate status in T2D patients receiving insulin treatment [320]. Neither study measured MMA. 

In a systematic review of six RCTs, including the two previously described, metformin use significantly lowered serum B12 in T2D patients in a dose-dependent manner [137]. Another systematic review and meta-analysis on the effect of metformin on Hcy levels was inconclusive [138]. Given the limited number of studies that have used MMA as a measure of vitamin B12 status, none have been included in a systematic review to date.

Data from survey and case-report studies indicate that metformin use may reduce the intestinal absorption of dietary B12 [139,321]. Other studies have observed clinical symptoms of B12 deficiency with long-term metformin use, including megaloblastic anemia [139,322] and peripheral neuropathy [323,324]. The process of B12-intrinsic-factor absorption is calcium-dependent, and metformin is known to affect calcium-dependent membrane action. To examine this relationship, a calcium supplementation study was conducted in T2D patients who were on metformin for three months prior [140]. Supplementation with 1.2 g/d calcium for one month was shown to reverse the observed metformin-induced malabsorption of B12, demonstrated by increased serum B12 and holotranscobalamin levels when compared with controls. 

In summary, observational and intervention studies have shown that metformin use may negatively affect vitamin B12 status in a duration- and dose-dependent manner through impaired intestinal absorption. Individuals already at risk of low B12 status, including the elderly and vegetarians, may be at greater risk during drug therapy. Although more studies that include functional markers of B12 status are needed, the current evidence is sufficient to recommend periodic assessment of vitamin B12 in patients taking metformin [325]. Furthermore, the concomitant use of a multivitamin with metformin appears to protect against B12 deficiency [326].

### 2.8. Oral Hypoglycemics: Thiazolidinediones (TZD)

#### Calcium and Vitamin D

There is consistent evidence that T2D patients have a higher incidence of bone fractures compared to non-diabetic patients, despite no significant differences in BMD between the two groups [327,328]. The use of TZD to improve insulin sensitivity in T2D patients has been shown to further increase the risk of fracture and decrease BMD [141,142,143]. TZD affect mesenchymal stem cells to the extent that adipogenesis increases and osteoblast formation decreases [329,330]. A number of trials have investigated the effect of TZD on BMD, bone turnover, and fracture risk, and collective findings from systematic reviews and meta-analyses indicate TZD treatment results in modest bone loss that may be specific to women [144]. Similarly, long-term TZD use has been shown to increase the risk of bone fracture in women, who are already at a higher risk for osteoporosis, bone loss, and bone fracture than men [145]. Furthermore, the intake of nutrients critical for bone health, including calcium, vitamin D, and magnesium, was reported to be insufficient in T2D patients on antidiabetic therapies [331].

In summary, although evidence indicates that TZD increase the risk for bone fracture and osteoporosis, in particular among older women, no studies have examined the potential protective effect of concurrent supplementation with calcium and vitamin D on bone health. Considering the dietary intake of these nutrients is likely insufficient in this population, supplementation may be warranted in some cases.

### 2.9. Oral Corticosteroids

#### 2.9.1. Calcium and Vitamin D

Extensive evidence from cross-sectional and longitudinal studies indicates that prior and current exposure to glucocorticoids increases the risk for bone loss and fractures [146]. A meta-analysis of 89 studies found a significant, dose-dependent relationship between glucocorticoid use at ≥5 mg/d and loss of BMD and increased fracture risk in the hip and spine, independent of age, gender, and effects of the underlying disease [147]. The risk of fracture increased rapidly during the first three to six months of treatment, and reportedly decreased after cessation of the drug.

Glucocorticoids decrease the number and function of osteoblasts [332,333] leading to an imbalance in bone formation and resorption, favoring bone loss [334,335]. Glucocorticoids also have an inhibitory effect on renal and intestinal calcium absorption by suppressing the transcription of calcium-transport genes, thus opposing the actions of vitamin D [335,336,337,338,339]. Decreased calcium absorption with corticosteroid use was confirmed in a short-term study of healthy adults taking 20 mg/d prednisone for 14 days, while circulating vitamin D levels were unaffected [148,340]. Additionally, most patients with glucocorticoid-induced osteoporosis do not have significantly elevated PTH levels compared to controls, suggesting that hyperparathyroidism is not a central or significant factor in this relationship [334].

Several RCTs have been conducted to determine the efficacy of calcium and vitamin D supplementation for the prevention of bone loss, fractures, and osteoporosis during glucocorticoid treatment. Overall, results have been mixed due to significant heterogeneity in the age and condition of the study populations, as well as in corticoid and supplement dose, type, duration, and study design [149,150,151,152,153]. However, in a meta-analysis of five RCTs, supplementation with calcium plus vitamin D had a significant effect on preventing bone loss at the lumbar spine and forearm, but not on femoral neck bone mass, fracture incidence, or bone resorption [154]. The combination of calcium plus vitamin D was also found to be more effective than calcium supplementation alone. While it is likely that calcium plus vitamin D supplementation does protect against bone loss in patients taking glucocorticoids, it may be insufficient to protect against bone fracture and osteoporosis. Supplementation is more effective when taken in conjunction with pharmacological treatments, such as bisphosphonates, which have been shown to be beneficial in reducing the risk of bone fractures and bone loss in corticosteroid users [341].

In summary, glucocorticoids have a negative effect on bone loss and fracture and are a leading cause of secondary osteoporosis, particularly in individuals who cannot achieve the recommended intake of calcium and vitamin D from diet alone, and in those who are otherwise at high risk of bone fractures and osteoporosis, e.g., advanced age or postmenopausal females [342,343]. Concurrent supplementation with calcium and vitamin D may be appropriate for some patients.

#### 2.9.2. Sodium and Potassium

Corticosteroid use has been reported to cause sodium and water retention as well as increased potassium excretion, potentially leading to hypertension [344,345,346,347]. The mechanism underlying this effect is not completely understood. Most of the current data on the potential transcriptional and post-translational regulatory effects of corticoids on sodium transport and absorption in different parts of the kidney are limited to in vitro and rodent studies, and are beyond the scope of this review [348]. A number of studies also indicate that glucocorticoids may induce hypertension through mechanisms independent of sodium retention and potassium excretion [345]. In general, it is recommended that patients on chronic corticosteroid therapy limit their sodium intake and monitor their potassium intake. A diet rich in potassium is likely sufficient to maintain normal levels during corticosteroid treatment, but potassium supplements may be recommended for individuals who are unable to obtain the recommended amount through diet alone [349].

#### 2.9.3. Chromium

A study investigating the effect of corticosteroid treatment on chromium status in 13 patients reported increased excretion of this essential mineral after three days of treatment, although the clinical significance is unclear [350]. In this same study, chromium supplementation of three patients with steroid-induced diabetes was found to improve fasting blood glucose levels. To date, no additional RCTs have been performed to validate these preliminary findings.

### 2.10. Bronchodilators: Beta2-Agonists and Inhaled Corticosteroids [ICS]

#### Calcium and Vitamin D 

Human studies investigating the effect of beta2-agonists on bone health are limited. In one population-based, case-control study an increased risk of hip and femur fractures was reported for higher doses, which was attenuated after adjusting for oral glucocorticoid use and underlying disease [351]. This finding is consistent with the results of a two-year randomized trial of ICS versus non-corticosteroid treatment on BMD in mild asthmatics taking beta2-agonists [352]. In this study, ICS dose was negatively associated with lumbar spine bone density, and no association was observed for the non-steroid group. 

The effects of long-term (≥12 months) ICS use on bone are currently unclear. A quantitative systematic review reported that ICS use may affect markers of bone metabolism and BMD in patients with asthmatic and chronic obstructive pulmonary diseases (COPD), as well as healthy adults [155]. This is similar to the findings of another meta-analysis in which a significant relationship between higher doses of ICS and increased bone turnover in asthmatic and mild COPD patients was observed [156], although at lower doses this relationship was no longer significant, and no association between ICS use and fracture risk was observed in either of these studies. An industry-sponsored meta-analysis reported BMD loss with long-term ICS use among patients with asthma and COPD did not differ from healthy controls [353].

A more recent systematic review and meta-analysis in which COPD patients were excluded found no significant associations between long-term ICS use, BMD, and fracture risk in asthmatic children and adults [157]. Other factors that negatively impact bone formation and would likely have an effect on BMD in COPD patients include the high prevalence of smoking, as well as low-grade systemic inflammation and cachexia [157,354]. Indeed, meta-analyses on the association between long term ICS use and fracture risk in COPD patients have only found a modest but significant, dose-dependent increase [158,159].

In summary, the effect of long-term ICS use may negatively influence bone metabolism and BMD in certain patients. The association appears to be stronger in COPD compared to asthmatic patients since the former are already at an increased risk of impaired bone health. No studies have tested the effect of calcium and vitamin D supplementation in ICS users on markers of bone health, and such studies are needed. Currently, only 17% of ICS users >50 year of age reported taking calcium and vitamin D supplements [355].

### 2.11. Antidepressants

#### Calcium and Vitamin D

Numerous studies have reported a significant association between the use of selective serotonin reuptake inhibitors (SSRIs) and risk of osteoporosis, with considerable evidence indicating these drugs increase the risk of fracture in a dose- and duration-dependent manner [168,169,170]. Additional evidence indicates SSRIs may also decrease BMD [315]. However, collective findings from cohort and case–control studies report the increased risk of fracture from SSRIs may be independent of BMD [356]. The mechanism of action underlying this relationship is not fully elucidated, although studies have shown serotonin receptors are present on osteoblasts, osteoclasts, and osteocytes, indicating SSRIs may influence bone formation and resorption through the activation of serotonin receptors [357].

Given the consistent evidence, studies examining the efficacy of calcium and vitamin D supplementation in conjunction with SSRI use on BMD and fracture risk are warranted.

### 2.12. Oral Contraceptives (OC)

#### 2.12.1. Vitamin B6

Tryptophan metabolism, an indirect measure of vitamin B6 status, is abnormal in OC users compared to controls and can be corrected with supplemental doses of vitamin B6 [358,359,360,361,362,363,364]. However, estrogens may influence tryptophan metabolism independently of B6 status [172,364], thus other markers of vitamin B6 levels, such as plasma 5′-phosphate (PLP), urinary 4-pyridoxic acid (4-PA), urinary B6, and erythrocyte aminotransferase or transaminase activity, should be used when studying the potential interaction between OCs and vitamin B6. OC users were observed to have significantly lower PLP in both fasting and non-fasting plasma compared to nonusers [365,366]. However, not all studies accounted for subjects’ dietary intake of vitamin B6, which can influence PLP levels.

The potential effects of OC use on vitamin B6 status have also been assessed in controlled, depletion–repletion feeding studies. In these studies, OC users and nonusers were fed a vitamin B6 deficient diet for one menstrual cycle, and then given a replete diet with 0.8–16.6 mg/d pyridoxine for another cycle [363,364]. A comprehensive array of markers was used to assess B6 status weekly. Among these markers, only tryptophan metabolites differed significantly between the OC users and nonusers. During the depletion period the decline in all parameters was similar between groups, and during repletion 1.8 mg pyridoxine was equally effective in increasing parameters of B6 status in both groups. Thus, vitamin B6 requirements were not higher in OC users, which was confirmed in a separate but similarly designed study [360,367].

When using erythrocyte transaminase as a biomarker of vitamin B6 status, one cross-sectional study of 233 women taking OCs found almost 50% of OC users, compared to 18% of nonusers, had marginal or deficient B6 status [368]. Conversely, in a study that analyzed both cross-sectional and longitudinal data of erythrocyte transaminase activity in young OC users vs. nonusers, no differences were reported [369].

In summary, results are mixed regarding the relationship between OC use and vitamin B6 requirements. Depending on the biomarker used to measure B6 status, OC use may negatively impact or have no effect on B6 levels, thus, widespread supplementation is not currently recommended [370]. However, intervention studies with vitamin B6 supplementation have reported improvements in clinical symptoms of B6 deficiency and fewer side effects in OC users who may be deficient in this vitamin [371,372]. 

#### 2.12.2. Vitamin B12

Several studies have consistently reported serum B12 levels are lower in OC users compared to nonusers [172,173,174,175,176,177,178,179,180,366], yet, no differences in Hcy and MMA have been observed [178,179,180,373,374]. While most of these studies adjusted for confounders, not all adjusted for dietary intake. The observed decrease in serum B12 and lack of a difference in Hcy and MMA among OC users may indicate a “redistribution” of B12 rather than a depletion [178]. Given the influence of OCs on circulating protein levels, it is possible they alter circulating B12 binding proteins, i.e., transcobalamins (TC). One study determined serum TC1, which circulates serum B12 but does not deliver it to tissues, was lower in OC users compared to nonusers, indicating a lower binding capacity of vitamin B12 in serum rather than a deficiency [179].

In summary, although there is consistent evidence that OC use is related to lower serum B12 levels, it is not clear whether this is actually indicative of a biochemical B12 deficiency. For populations already at risk for B12 deficiency, such as vegetarians, it is unclear how a potential change in serum B12 binding capacity due to OC use may affect them.

#### 2.12.3. Folate

The earliest evidence for a negative effect of OC use on folate status was reported in the 1960s and 70s. In addition to several case studies in which folate deficiency and megaloblastic anemia were reported in OC users admitted to the hospital [181,375,376,377], a cross sectional analysis of women taking OCs for two months to five years found significantly lower mean serum folate levels compared with controls that also appeared to be associated with duration of use [182]. These findings were later confirmed, although in this study folate status was not related to the duration of OC use [378]. Other studies have found the absorption of folate polyglutamates, but not monoglutamate, to be lower in OC users [181], and that OC use may be related to increased metabolism and urinary excretion of folate [379]. 

A recent meta-analysis that included case–control, cohort studies, and clinical trials from 1970–2013 concluded that OC use is, indeed, associated with lower blood folate status [183], though other studies conducted during the same time period found no difference in folate status between OC users and controls [380,381,382]. This discrepancy may be due to confounding issues, including lack of adjustment for dietary folate intake, supplement use, smoking, and alcohol consumption [172,383]. Even in studies that considered dietary intake of folate, results varied [374,381,384]. It is possible inter-individual genetic differences in folate handling may contribute to the variation [172].

In summary, the collective scientific evidence, at present, is too inconsistent to support the conclusion that OC use causes folate deficiency. However, maintaining normal folate status is critical among women of child-bearing age, regardless of OC use. In 2010, the Food and Drug Administration approved a folate-containing OC for use. Two recent clinical trials tested the effects of this folate-fortified product, and both reported clinically significant increases in markers of folate status when compared to women given an OC absent folate [385,386].

#### 2.12.4. Calcium

Several studies have reported that OC use may increase BMD in women ranging in age from young adulthood to perimenopause [184,185,186,187]. OC use may decrease bone turnover as well as both 2 h renal calcium excretion, a marker of bone-derived calcium loss, and 24 h renal calcium excretion, a marker of total calcium loss (both dietary and bone-derived) [387,388,389]. In contrast, others have reported a detrimental effect of OC use on BMD. Although multiple factors must be considered when determining the effect of OC on BMD, including age at first use [188] and race [189], physical activity appears to be a major factor in this relationship. A cross-sectional study conducted in women age 20–35 year reported those with the highest BMD values were characterized as having combined long-term exercise and short-term OC use, while no relationship was observed with long-term exercise and long-term OC use [190]. An intervention study in which OC users and nonusers were randomized into exercise or non-exercise groups for two years found total body bone mineral content (TBBMC) was higher in the exercise group and lower in OC users, but either exercise or OC use alone suppressed the normal accretion of bone mass and mechanical strength at the femoral neck, and the combination of exercise plus OC use had a less suppressive effect on the latter measures [191,192]. A follow-up study to investigate the effect of a one-year dietary calcium intervention at either <800, 1000–1100, or 1200–1300 mg/d on BMD in 18–30 years old OC users vs. nonusers, found that both medium and high intake of calcium from dairy products protected OC users from total hip and spine BMD loss when compared with those in the low calcium intake group [193]. No studies have tested the effect of calcium supplements on bone health in OC users.

In summary, the effect of OC use on TBBMC and BMD at specific sites may depend on a number of factors, including type and level of physical activity as well as calcium intake. Women on long-term OC therapy and high physical activity may be at highest risk.

#### 2.12.5. Magnesium

Most cross-sectional studies have shown serum magnesium levels are lower in OC users compared to both nonusers and women on other forms of contraception [194,195,196,197,198], with one exception [199]. An increase in the blood calcium to magnesium ratio due to low magnesium levels can influence blood coagulation processes [390,391]. Indeed, a systematic review and network meta-analysis of 26 observational studies that investigated the risk of venous thrombosis for different combined OCs concluded OC use increased the risk of venous thrombosis, and effect size depended on the combination used [200].

Further studies are needed to investigate whether the effects of dietary or supplemental magnesium can influence the calcium: magnesium ratio and the potential for blood clotting in OC users.

#### 2.12.6. Vitamin C and E

While some studies indicate circulating vitamin C levels are lower in OC users compared to nonusers [383,392,393], others indicate little threat to individuals who live a healthy lifestyle and consume a diet adequate in vitamin C [175,394]. Then again, several studies show chronic OC use leads to increased oxidative stress, in particular lipid peroxidation, and lower circulating vitamin E [394,395,396,397,398,399,400]. Enhanced oxidative stress and lipid peroxidation may represent a potential risk for cardiovascular disease. One study reported an increase in catalase and glutathione peroxidase activities, i.e., endogenous antioxidant defenses, in 19 young, healthy, non-smoking women after nine cycles of OC use when compared to baseline levels [401]. In a larger, controlled study of 120 healthy women, age 18–40 years, OC users were randomized to receive either supplements with 150 mg vitamin C and 200 IU vitamin or no supplements, and compared with non-supplemented, non-OC users. After four weeks, increased plasma malondialdehyde levels, a marker of lipid peroxidation, and reduced glutathione peroxidase and reductase activities were reported in the non-supplemented OC users, while the directions of each were reversed in the supplemented OC users when compared with the control group [395].

More work is needed to fully elucidate the potential mechanisms through which OC use affects antioxidant balance, particularly since there may be potential implications for cardiovascular disease risk.

## 3. Part II: Medications Potentially Affected by Nutritional Status

### 3.1. Antidepressants and Folate

Several observational studies report lower folate status, as measured by serum, plasma, and red blood cell folate concentrations or plasma Hcy, in patients with major depressive disorder (MDD) compared to healthy controls [402,403,404]. Additionally, genotyping analysis determined gene variants of methyltetrahydrofolate (MTHFR) are associated with risk of being diagnosed with depression, findings that were confirmed in a meta-analysis of similar studies [160]. Analyses of red blood cell folate from a population of young adults in the U.S. (age 15–39 years) and dietary folate intake in middle-aged Finnish men (age 42–60 years) found both measures were inversely associated with depressive symptoms in these cohorts, even after adjusting for a comprehensive number of confounding variables [405,406]. Interestingly, in older adult populations the correlation between folate status and depressive symptoms was not found to be significant [407,408]. 

RCTs to investigate the effects of including a folate supplement with antidepressant treatment, specifically SSRI, have been conducted. In one study 127 patients with MDD and mean age of 43 years were randomized to receive either 500 µg folic acid or placebo in addition to 20 mg SSRI daily for 10 weeks [161]. In this study, women supplemented with folic acid had significantly higher plasma folate, lower Hcy, greater improvements in depressive symptoms on the Hamilton Rating Scale, and fewer reported side effects when compared to the placebo group, while no significant effects were reported in men.

The relationship between folate status and depressive symptoms is of interest because, despite the widespread use of antidepressants among patients with MDD, 31–49% of patients are either partial- or non-responders [409]. Additionally, about one-third of patients classified as responders to antidepressants were reported to have cognitive or physical symptoms, likely to be residual symptoms of depression, side effects of medication, or both [410]. Furthermore, observational studies of middle aged and older adults found low blood folate levels were associated with greater resistance to improvement following antidepressant therapy [162,163,164].

In a multicenter, sequential parallel comparison design trial 75 outpatients with SSRI-resistant MDD given 15 mg/d l-methylfolate adjunctive to their current SSRI dose for 1–2 months showed significantly greater efficacy in the response rate and degree of change in depression symptom score, as well as a select measure of symptom severity compared to those given a placebo in addition to their SSRI [165]. The authors reported in a prior trial of 148 patients given half the amount of l-methylfolate (7.5 mg/d) that no significant outcomes were observed. The higher folate dose was also well tolerated and, thus, may be an effective and safe treatment strategy for MDD patients with partial or no response to SSRI treatment. Moreover, retrospective analyses of adults given l-methylfolate in addition to antidepressants reported the combination was more effective in improving depression symptoms than antidepressant monotherapy [166,167]. 

In summary, the evidence suggests low folate status may be associated with depression and adjunctive treatment with folate may benefit individuals on antidepressant therapy, including patients with MDD who are nonresponsive to antidepressant medication. Currently there are no official guidelines regarding the most effective form, dose, or duration of folate supplementation in this capacity.

### 3.2. ACE Inhibitors and Iron

The most common side effect of chronic ACE inhibitor use is a dry cough, which occurs in 5–39% of patients [411]. Given the evidence that nitric oxide synthesis is down-regulated in the presence of iron [412], it has been hypothesized that nitric oxide generation in bronchial epithelial cells may contribute to the ACE-inhibitor-induced cough. To test this, a placebo-controlled RCT was performed to determine the effect of supplementation with 256 mg/d ferrous sulfate for four weeks in 19 patients on ACE inhibitors who had developed dry cough [413]. Following iron supplementation, the reduction in mean score of daily cough severity was significantly greater when compared with the placebo group, and three subjects in the iron group reported complete cough resolution. No changes in iron status were observed in either group. These findings suggest that iron supplementation may ameliorate ACE-inhibitor-induced cough, although additional research is needed to confirm.

## 4. Discussion

A summary of the evidence related to potential drug–nutrient interactions with use of the most often prescribed medications for commonly diagnosed conditions among U.S. adults—including PPIs, NSAIDs, anti-hypertensives, hypercholesterolemics, oral hypoglycemics and corticosteroids, bronchodilators, SSRI antidepressants, and OCs—was presented (Table 1). Briefly, PPI use may compromise B12 status, particularly in older adults and those with *H. pylori* infection, further impair iron status in anemic patients, and increase bone fractures in high risk individuals. PPI use may also reduce the absorption of magnesium, zinc, and beta-carotene, although evidence for the latter is limited and the clinical implications are unclear. Aspirin alters vitamin C absorption in leukocytes, and may lower iron status in older adults, particularly those with *H. pylori* infection. Interestingly additional vitamin C may prevent aspirin-induced gastric lesions. Loop diuretics enhance calcium excretion, and may impair calcium balance in older adults. Their use is also associated with poor thiamin status. Thiazides, on the other hand, may not negatively affect calcium status, but both types of diuretic can lower magnesium and lead to hypokalemia due to increased urinary potassium excretion. In addition, thiazide use may lead to tissue zinc depletion. ACE inhibitors, captopril in particular, may also impair zinc status, an issue of concern in older adults and other susceptible individuals. Statin use lowers serum CoQ10, especially in older adults, and may negatively impact the status of the fat-soluble vitamins, D, E, and beta-carotene. Metformin impairs B12 status in susceptible individuals, while concurrent supplementation with this vitamin may prevent deficiency. Supplementation with calcium and vitamin D combined may reduce the risk of fractures and bone loss with glucocorticoid use. The use of bronchodilators and SSRI antidepressants may also compromise bone health, although no studies have examined the potential effects of concurrent calcium and vitamin D with either drug. In some women, improved calcium status may protect against bone loss with OC use. OC may also compromise B-vitamin status, lower serum magnesium levels, and increase markers of oxidative stress, the latter of which may be reduced with the antioxidant vitamins C and E.

For the majority of the interactions described in this paper, more high-quality intervention trials are needed to better understand their clinical importance and potential consequences. A number of these studies have identified potential risk factors that may make certain populations more susceptible, but guidelines on how to best manage and/or prevent drug-induced nutrient inadequacies are lacking. Although widespread supplementation is not currently recommended, it is important to ensure at-risk patients reach their recommended intakes for essential vitamins and minerals.

There is no doubt the use of commonly prescribed medications that can adversely impact nutritional status is on the rise [414]. Given the increasing prevalence of conditions that require long term medication use, and an inadequately nourished adult population, the potential public health implications are profound [415,416,417]. While young and middle-aged adults are certainly affected, there is particular concern for older adults who are more likely to use multiple concurrent medications. Aging adults also undergo physiological changes that affect nutrient needs, and their ability to meet these needs, which may further compound the issue.

Ideally, clinicians should recommend their patients consume a sufficient quantity and variety of nutrient-dense foods in their daily diet, i.e., vegetables, fruits, beans and legumes, nuts and seeds, whole grains, fish, lean proteins and dairy or fortified alternatives such as soy milk, in order to meet their calorie and nutrient needs, and limit their intake of high, but empty, calorie foods and beverages. Unfortunately, health professionals are not routinely trained to provide dietary advice, nor do they have time to provide effective counseling during an office visit. Moreover, public health campaigns designed to promote healthy eating patterns are often met with a modicum of success manifested only after prolonged exposure.

Many adults are aware of potential shortfalls in their diet and take a dietary supplement, most often a multivitamin/mineral (MVM) preparation, to compensate [418,419,420]. Indeed, observational data indicates adults who take a full spectrum MVM supplement are less likely than nonusers to be deficient in any micronutrient (14% vs. 40%, *p* < 0.02) [421]. Intervention studies confirm MVM supplementation helps fulfill micronutrient requirements and improve nutritional status even in healthy adults [422,423]. A growing body of evidence also supports the use of MVM in preventing certain chronic conditions, especially with long term use [424,425,426,427,428]. Historically, physicians have been hesitant to recommend a MVM, despite little evidence of harm [429,430,431]. Current evidence indicates age- and gender-appropriate MVM supplements, formulated at or near 100% of the daily value for most micronutrients, are generally well tolerated and do not appear to increase the risk of mortality or disease [419].

In conjunction with an overall healthy diet, a single daily MVM may be a practical and efficacious way to maintain or improve micronutrient status in patients at risk of deficiencies, such as those taking medications known to compromise nutritional status. Practitioners should be aware that a single dose MVM will not provide sufficient calcium, CoQ10, or fish oil. Depending on the medication in use, as well as the patient’s baseline status, usual dietary intake, and current condition, a separate supplement may be warranted.

## 5. Conclusions

When managing drug–nutrient interactions, it is essential to consider the strength and quality of the available evidence. Even though the importance of these interactions has long been recognized, appropriately designed observational and intervention studies examining the role of dietary intervention and/or supplementation in ameliorating the effects of chronic medication use are lacking. The summary evidence presented in this review will help inform future research efforts and, ultimately, guide recommendations for patient care.

## Figures and Tables

**Table 1:** Summary of potential drug–nutrient interactions and known risk factors.

Drug Category	Name	Nutrient	Effect on Nutrient Status or Function	Human Studies ^1^	Risk Factors	References
Acid-Suppressing Drugs	Proton Pump Inhibitors	Vitamin B12 Vitamin C Iron CalciumMagnesium Zinc β-Carotene	Decrease Decrease Decrease Decrease Decrease Decrease Decrease	5 observational 5 intervention 1 observation 4 intervention 2 case reports 1 observational 2 intervention >10 observational 4 intervention 30 case reports 2 intervention 1 intervention	Advanced age *H. pylori* infection Genetics (slow metabolizers Low dietary intake (vegetarians) *H. pylori* infection Pre-existing iron deficiency Vegetarians Advanced age Women Advanced age Duration of drug use Women Undetermined Undetermined	[10,11,12,13,14,15,16,17] [18,19,20,21,22] [23] [24,25,26,27,28] [29,30,31] -- --
Non-Steroidal Anti-Inflammatory Drugs	Aspirin	Vitamin C Iron	Decrease Decrease	1 observational 4 intervention 6 observational 8 intervention	Absence of cold virus Advanced age *H. pylori* infection	[32,33,34,35] [36,37,38,39]
Anti-Hypertensives	Diuretics (loop, thiazide) Diuretics (potassium-sparing) Angiotensin-Converting Enzyme Inhibitors Calcium Channel Blockers	Calcium Magnesium Thiamin Zinc Potassium Folate Zinc Potassium Iron ^2^ Folate Potassium	Decrease (loop) Increase (thiazide) Decrease (loop and thiazide) Decrease (loop) Decrease (thiazide) Decrease (thiazide) Decrease Decrease Increase N/A Decrease Increase	>20 observational 9 intervention >10 observational 1 intervention 4 observational 2 intervention 3 observational 6 intervention 3 observational >100 intervention 2 case reports 1 observational 3 observational 6 intervention 2 case reports 3 observational 1 intervention 1 intervention 6 case reports 3 observational 2 case reports 2 observational	Dose/duration of drug use Form of loop diuretic Advanced age Women Advanced age Heart failure Low Mg intake Alcohol use Long-term use Coronary heart failure Advanced age Low dietary thiamin intake Hepatic cirrhosis Diabetes mellitus Heart failure Gastro-intestinal disorders Renal disease Low dietary zinc intake Dose Form of thiazide used Low folate status Impaired liver function Liver cirrhosis (alcoholics) Use of captopril Heart failure Renal disease Age (elderly) Renal disease Diabetes mellitus Congestive heart failure Potassium supplement use Undetermined Presence of dental plaque Poor oral hygiene Gender (men) Dose Low folate intakes Concurrent use of beta-blockers	[40,41,42,43,44,45,46,47,48] loop [42,49,50,51,52,53,54] thiazide [55,56,57] [58,59,60,61,62] [63,64,65,66,67,68,69] [70,71,72,73,74] [75,76] [77,78,79,80,81,82,83,84,85] [86,87,88,89,90,91] -- [92,93,94,95,96,97,98,99] [100,101,102,103]
Hypercholesterolemics	Statins	Coenzyme Q10 Vitamin D Vitamin E/β-Carotene	Decrease Increase/Decrease Increase/Decrease	7 observational >10 intervention >10 observational 4 intervention 1 observational 6 intervention	Dose Advanced age Statin-associated myopathy Heart disease Vitamin D deficiency Statin-associated myopathy Undetermined	[104,105,106,107,108,109,110,111,112,113,114] [115,116,117,118,119,120,121,122,123,124,125,126,127,128] --
Hypoglycemics	Biguanides (Metformin) Thiazolidinediones	Vitamin B12 Calcium/Vitamin D	Decrease Decrease	>10 observational >10 intervention 3 observational >10 intervention	Dose/duration of drug use Advanced age Vegetarians Advanced age Women Low calcium/vitamin D intake	[129,130,131,132,133,134,135,136,137,138,139,140] [141,142,143,144,145]
Corticosteroids	Glucocorticoids (oral)	Calcium/Vitamin D Sodium/Potassium Chromium	Decrease Increase (sodium) Decrease (potassium) Decrease	>80 observational >10 intervention ~5 case reports/observational 1 intervention 1 intervention	Low calcium/vitamin D intake At risk for bone fracture/loss Undetermined Undetermined	[146,147,148,149,150,151,152,153,154] -- --
Bronchodilators	Corticosteroids (inhaled)	Calcium/Vitamin D	Decrease	>10 observational >10 intervention	Presence of COPDSmoking At risk for bone fracture/loss Low calcium/vitamin D intake	[155,156,157,158,159]
Antidepressants	Selective Serotonin Reuptake Inhibitors	Folate ^3^ Calcium/Vitamin D	Increase ^3^ Decrease	5 observational 2 intervention >10 observational	Low folate intake Genetics (MTHFR variants) Alcoholism At risk for bone fracture/loss Low calcium/vitamin D intake	[160,161,162,163,164,165,166,167] [168,169,170,171]
Oral Contraceptives	Estrogen and/or Progesterone	Vitamin B6 Vitamin B12/Folate Calcium Magnesium Vitamin C/Vitamin E	Decrease Decrease Increase/decrease Decrease Decrease	>10 observational 5 intervention 4 case reports >30 observational 5 intervention 7 observational 6 intervention >20 observational >10 observational 2 intervention	Undetermined Vegetarians Low folate intake Genetics (folate) Duration of drug use Duration of drug use Physical activity level Low calcium intake Age at first use Race Type of combined OC used Undetermined	-- [172,173,174,175,176,177,178,179,180,181,182,183] [184,185,186,187,188,189,190,191,192,193] [194,195,196,197,198,199,200] --

^1^ Total number of studies that have investigated the potential drug–nutrient interaction (includes both significant and null results); ^2^ Nutrient effect on drug side effect; ^3^ Effect of nutrient on drug efficacy.
